# Periportal Stellate Cells in Subjects with Chronic Hepatitis C with a Varied Serum Alanine Aminotransferase Level

**DOI:** 10.1186/1476-5926-2-S1-S15

**Published:** 2004-01-14

**Authors:** Mutsunori Fujiwara, Kazuhiko Besshi, Tamiko Takemura, Yukiko Ito, Takeshi Tsujino, Michiko Yamagata, Nobuyuki Minagawa, Ryo Nakata, Michiyasu Yoshitsugu, Yoshihisa Kato, Masashi Ihori, Isao Okayasu, Haruki Senoo, Kenjiro Wake

**Affiliations:** 1 Division of Pathology, Japanese Red Cross Medical Center, Hiroo, Shibuya-ku, Tokyo, Japan 150-8935; 2 Department of Internal Medicine, Japanese Red Cross Medical Center, Hiroo, Shibuya-ku, Tokyo, Japan 150-8935; 3 Drug Safety Research Laboratory, Taiho Pharmaceutical Co., Ltd., Japan; 4 Department of Pathology, School of Medicine, Kitasato University, Japan; 5 Department of Anatomy, Akita University School of Medicine, Japan; 6 Department of Anatomy, School of Medicine, Tokyo Medical and Dental University, Japan

## Introduction

Recently it has been reported that HCV-related cirrhotic patients with persistently high serum levels of alanine aminotransferase (ALT) activity have higher risk of development of hepatocellular carcinoma (HCC) than those with persistently low levels of ALT activity [1]. Vitamin A has been demonstrated to have many biological functions in regulation of growth and differentiation of normal and cancer cells. Hepatic stellate cells (SCs) are the main storage site of vitamin A and regulate homeostasis of vitamin A. To analyze cellular and molecular mechanism in the relationship between persistent high serum level of ALT and HCC development from the viewpoint of vitamin A handling, this study was performed.

## Methods

Liver biopsy specimens obtained from 61 patients with chronic hepatitis C, demonstrated to have antibody to HCV by ELISA method, were investigated light and electron microscopically. They were subdivided into 4 groups according to their serum ALT levels. Group A was comprised of 21 patients whose serum ALT levels were persistently normal (–40 IU/l) for at least one year: 6 men and 15 women, 29 to 64 years old. Group B was comprised of 23 patients whose serum ALT levels varied from –80 IU/l to –40 IU/l : 14 men and 9 women, 27 to 69 years old. Group C was comprised of 9 patients whose serum ALT levels varied from –80 IU/l to &lt;80 IU/l – >40 IU/l : 8 men and one woman, 32 to 65 years old. Group D was comprised of 8 patients whose serum ALT levels were persistently high (–80 IU/l) for at least one year : 6 men and 2 women, 29 to 63 years old.

**Figure 1:**
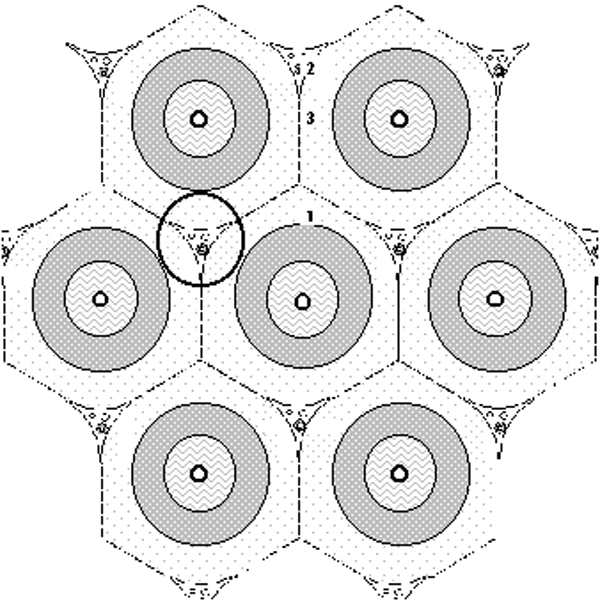
Lamers' model of acinar zonation. "The periportal area" roughly corresponds to acinar zone 1.

**Figure 2:**
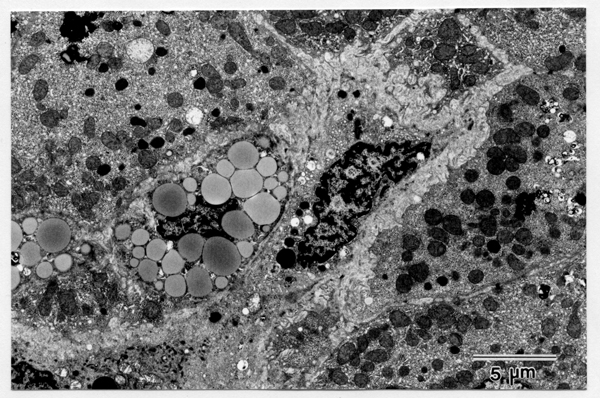
Periportal vitamin A-rich stellate cell which contains a number of lipid droplets and has indented nucleus. Bar = 5 micrometers.

One micrometer-thin sections from tissues for electron microscopy were stained with methylene blue and viewed to count periportal vitamin A-rich or -poor SCs/mm^2^. The periportal area (Figure 1) roughly corresponds to acinar zone 1 proposed by Lamers and his colleagues [2]. Ultrathin sections were stained with uranyl acetate and lead citrate and examined with an electron microscope (JEM-100CX). Vitamin A-rich SCs were defined as the SCs containing more than 10 vitamin A droplets whose diameters are more than one micrometer (Figure 2). Cells showing morphological features of fibroblasts or fibrocytes with few or no vitamin A droplets were not added to total SCs.

All results were given as the mean – standard deviation, and the Student's t-test was used for statistical analysis.

## Results

The number of periportal vitamin A-rich SCs was significantly smaller in group D with persistently high serum levels of ALT than those with persistently low serum levels of ALT (p &lt; 0.05) (Table 1). The total number of periportal vitamin A-rich or -poor SCs was smaller in group D than in group A (p &lt; 0.01) or group B (p &lt; 0.05).

**Table 1:** Periportal Stellate Cells (SCs) in Subjects with Chronic Hepatitis C with fluctuation of Serum ALT Level

Group	N	Age (years)	The number of periportal vitamin A-rich SCs (/mm^2^)	The total number of periportal vitamin A-rich and -poor SCs (/mm^2^)
A	21	48.1 – 11.4	13.3 – 8.3	63.0 – 24.2
B	23	47.6 – 13.1	15.0 – 15.3	57.0 – 25.5
C	9	49.8 – 12.1	14.2 – 12.1	55.0 – 30.9
D	8	49.1 – 12.3	5.3 – 9.5 a	34.1 – 17.4 aa,b

Significant difference compared with group A: a) p &lt; 0.05, aa) p &lt; 0.01 Significant difference compared with group B: b) p &lt; 0.05 No significant difference compared with group C

Portal fibrosis in group D is attributed to the increase in SCs if we include myofibroblasts or fibroblasts with few or no vitamin A droplets. However, a decrease in periportal SC was observed in this study because myofibroblast-like cells or fibroblasts with fewer vitamin A droplets were not added to total SCs.

## Discussion

Many studies suggest that persistent inflammation may be involved in development of carcinoma including HCC. Tarao, et al. [3] analyzed 28 patients with HCV-related cirrhosis who had persistently high ALT levels (–80 IU/l) and 28 patients who had persistently low ALT levels (&lt;80 IU/l), and revealed that 20 patients in the high ALT group developed HCC during an average observation period of 7.0 – 0.5 years. Of the 28 patients in the low ALT group, only 7 patients developed HCC during an average observation period of 8.7 – 0.6 years. They also reported a close correlation between multicentric hepatocarcinogenesis and sustained necroinflammation of the liver in patients with hepatitis C virus-associated cirrhosis, and suggested a strong correlation between the development of HCC and liver cell necrosis.

Our present study shows that the number of periportal vitamin A-rich SCs and vitamin A-poor SCs is significantly smaller in CH patients with persistently high serum levels of ALT (group D) than those with persistently low serum levels of ALT (group A). SCs in the patients with fluctuation of ALT levels (groups B and C), indicating that the inflammation was not persistently active, had no significant decrease in these numbers. This result also supports that vitamin A content in SCs decreases only when the necroinflammation is persistently active. This is the first report demonstrating that there is an explicit relationship between persistent active necroinflammation in the liver and depletion of vitamin A in SCs.
